# Association Between Salivary High-Sensitivity C-reactive Protein Concentrations and Clinical Stage of Periodontitis in an Indian Population: A Cross-Sectional Analytical Study

**DOI:** 10.7759/cureus.113835

**Published:** 2026-08-02

**Authors:** Beena George, Tanya Nandkeoliar, Neelam Das, Sajidali S Saiyad, Pooja Bhagwat, Shirish K Chokshi, Sonali Thakare

**Affiliations:** 1 Department of Oral Pathology and Microbiology, Sree Mookambika Institute of Dental Sciences, Kulasekharam, IND; 2 Department of Periodontology, Dental College, Regional Institute of Medical Sciences, Imphal, IND; 3 Department of Periodontology, Sri Sai College of Dental Surgery, Vikarabad, IND; 4 Department of Physiology, Pacific Medical College and Hospital, Pacific Medical University, Udaipur, IND; 5 Department of Oral Pathology and Microbiology, V.Y.W.S. Dental College & Hospital, Amravati, IND; 6 Department of Obstetrics and Gynaecology, Dr. N.D. Desai Faculty Of Medical Science and Research Centre, Dharmsinh Desai University, College Road, Nadiad, IND

**Keywords:** high-sensitivity c-reactive protein, hs-crp, inflammatory biomarkers, non-invasive diagnostics, periodontal disease staging, periodontitis, precision dentistry, saliva, salivary biomarkers

## Abstract

Background

Periodontitis is a chronic inflammatory disease associated with both local tissue destruction and systemic inflammatory responses. Salivary high-sensitivity C-reactive protein (hs-CRP) has emerged as a promising non-invasive biomarker, but its association with periodontal disease severity remains insufficiently investigated in Indian populations.

Objective

To evaluate the association between salivary hs-CRP concentrations and the clinical stage of periodontitis in an Indian population and to examine their relationship with established periodontal clinical parameters.

Methods

This institution-based cross-sectional analytical study included 150 adults equally allocated into periodontally healthy, Stage I-II periodontitis, and Stage III-IV periodontitis groups (n = 50 each). Comprehensive periodontal examinations were performed according to the 2017 World Workshop Classification, and unstimulated whole saliva samples were collected for hs-CRP estimation using a high-sensitivity enzyme-linked immunosorbent assay (ELISA). Group comparisons were performed using one-way analysis of variance(ANOVA) with Bonferroni post hoc analysis. Correlation analyses and multiple linear regression were conducted to evaluate associations between salivary hs-CRP concentrations and clinical periodontal parameters after adjustment for age, sex, and body mass index (BMI).

Results

Salivary hs-CRP concentrations increased significantly with advancing periodontitis stage (p < 0.001). Significant positive correlations were observed between hs-CRP and periodontal clinical parameters, particularly clinical attachment level and probing pocket depth. Periodontitis stage remained an independent predictor of salivary hs-CRP after multivariable adjustment.

Conclusions

Salivary hs-CRP concentrations increase progressively with advancing clinical stage of periodontitis and are independently associated with periodontal disease severity. These findings support the use of salivary hs-CRP as a simple, non-invasive adjunctive biomarker for periodontal assessment and provide contemporary evidence using the 2017 World Workshop Classification in an Indian population. Prospective multicenter studies are warranted to validate its clinical utility and integration into multimarker saliva-based diagnostic strategies.

## Introduction

Periodontitis is a chronic inflammatory disease characterized by gradual destruction of the periodontal tissues, leading to clinical attachment loss, periodontal pocket formation, alveolar bone resorption, and eventual loss of teeth [1]. Given its high prevalence in India and established associations with cardiovascular disease, diabetes mellitus, rheumatoid arthritis, and adverse pregnancy outcomes, reliable biomarkers that reflect inflammatory burden are needed [1-3].

C-reactive protein (CRP) is a well-established biomarker of systemic inflammation. High-sensitivity CRP (hs-CRP) assays enable detection of low-grade inflammatory responses with greater sensitivity than conventional assays [4]. Increasing evidence links periodontal inflammation with elevated CRP concentrations, supporting the role of periodontitis as a contributor to systemic inflammatory burden [3,4].

Saliva has emerged as a promising diagnostic biofluid because it can be collected non-invasively, repeatedly, and at low cost. Advances in salivary diagnostics have identified inflammatory biomarkers that reflect both local periodontal inflammation and systemic inflammatory responses, supporting the use of saliva for periodontal disease assessment and treatment monitoring [5,6].

Several studies have reported significantly higher salivary CRP concentrations in patients with periodontitis than in periodontally healthy individuals. An Indian study by Sawhney and Ralli demonstrated progressively increasing salivary CRP levels with disease severity, findings that have been corroborated by subsequent observational studies [7-9]. In addition, systematic reviews have shown that serum and salivary inflammatory biomarkers decline following successful periodontal therapy, highlighting their potential utility for assessing treatment response [3,5].

Despite growing evidence supporting CRP as a periodontal biomarker, important knowledge gaps remain. Most previous studies, including those from India, used the pre-2017 classification that distinguished chronic and aggressive periodontitis rather than the current staging system [7]. The 2017 World Workshop Classification provides a standardized framework based on disease severity and complexity, yet evidence evaluating salivary hs-CRP across these contemporary clinical stages remains limited, particularly in Indian populations [10]. Moreover, most available studies have focused on serum CRP, with comparatively few investigating salivary hs-CRP using the current classification system [5,6,11].

The primary objective of this study was to evaluate differences in salivary high-sensitivity C-reactive protein (hs-CRP) concentrations across the clinical stages of periodontitis defined by the 2017 World Workshop Classification in an Indian population. The primary outcome variable was salivary hs-CRP concentration. The secondary objective was to examine the relationship between salivary hs-CRP concentrations and established periodontal clinical parameters, including probing pocket depth, clinical attachment level, bleeding on probing, plaque index, and gingival index. We hypothesized a progressive (dose-response) increase in salivary hs-CRP concentrations with advancing periodontal disease stage, accompanied by positive correlations with measures of periodontal tissue destruction and inflammation.

## Materials and methods

Study design and setting

This investigation adopted an institution-based cross-sectional analytical design and enrolled adult participants attending the Department of Periodontology, Pacific Medical College, Udaipur, India. The primary objective was to evaluate the association between salivary high-sensitivity C-reactive protein (hs-CRP) concentrations and the clinical stage of periodontitis. The Institutional Ethics Committee of Pacific Medical College and Hospital approved the study protocol (Approval No. PMU/PMCH/IEC/GEN/2023/137; 27 June 2023). Written informed consent was obtained from all participants before enrolment. The study complied with the Declaration of Helsinki and is reported according to the STROBE guideline for observational studies [12].

Sample size calculation

The required sample size was determined a priori using G*Power software (Version 3.1.9.7; Heinrich-Heine University, Düsseldorf, Germany) [13]. Because no previous Indian study had evaluated salivary high-sensitivity C-reactive protein (hs-CRP) across the clinical stages of periodontitis defined by the 2017 World Workshop Classification, the expected effect size was derived from the salivary C-reactive protein concentrations reported by Sawhney and Ralli in a comparable Indian cohort [7]. The reported group means and standard deviations were used to calculate the standardized effect size (Cohen's f = 0.31), which served as the input parameter for one-way ANOVA (fixed-effects, omnibus test). Using a significance level of 5% (two-sided), statistical power of 80%, and equal allocation among three study groups, the calculated minimum sample size was 132 participants. Considering the possibility of sample loss during saliva collection, specimen processing, biochemical analysis, and data cleaning, the sample size was conservatively inflated by approximately 14%, resulting in a final recruitment target of 150 participants (50 participants per group).

Study participants

Consecutive adult patients presenting to the outpatient Department of Periodontology, Pacific Hospital, Udaipur, were screened for eligibility throughout the study period. Individuals aged 18 years or older, with a minimum of 20 natural teeth and willing to provide written informed consent, were considered eligible.

Study participants were assigned to one of three groups using the diagnostic criteria of the 2017 World Workshop Classification of Periodontal and Peri-Implant Diseases and Conditions [9]: (i) periodontally healthy, (ii) Stage I-II periodontitis, and (iii) Stage III-IV periodontitis.

Diagnosis and staging were performed according to the 2017 World Workshop Classification of Periodontal and Peri-Implant Diseases and Conditions [9]. This classification is publicly available and may be used for research with appropriate citation.

Individuals presenting with conditions known to alter inflammatory biomarker concentrations were excluded. These included diabetes mellitus, autoimmune disorders, chronic inflammatory diseases, cardiovascular disease, malignancy, acute infections or febrile illness within the preceding four weeks, confirmed COVID-19 infection during the previous four weeks, pregnancy or lactation, current use of smoked or smokeless tobacco, obesity (body mass index ≥30 kg/m²), ongoing immunosuppressive therapy, periodontal treatment within the previous six months, and administration of antibiotics or anti-inflammatory medications within the preceding three months.

Clinical periodontal examination

Comprehensive periodontal examinations were performed by a single calibrated examiner using a UNC-15 periodontal probe (University of North Carolina-15; Hu-Friedy, Chicago, IL, USA) under standardized clinical conditions. Examiner calibration was completed before participant recruitment using 10 volunteers who were not included in the study. Measurement reliability for Probing Pocket Depth (PPD) and Clinical Attachment Level (CAL) was assessed using the intraclass correlation coefficient (ICC), demonstrating excellent intra-examiner repeatability (ICC: 0.91 for PPD and 0.89 for CAL).

Each participant underwent a full-mouth periodontal examination at six sites per tooth (mesiobuccal, mid-buccal, distobuccal, mesiolingual, mid-lingual, and distolingual), excluding third molars. Clinical assessments included Plaque Index (PI), Gingival Index (GI), Bleeding on Probing (BOP), Probing Pocket Depth (PPD), and Clinical Attachment Level (CAL). Periodontal diagnosis and staging followed the 2017 World Workshop Classification of Periodontal and Peri-Implant Diseases and Conditions. Disease stage was assigned using clinical attachment loss, probing pocket depth, radiographic bone loss, tooth loss attributable to periodontitis, and disease complexity, based on combined clinical and radiographic findings [9]. Radiographic bone loss was assessed using digital intraoral periapical radiographs obtained during routine periodontal evaluation and incorporated into the final stage assignment.

The periodontal indices employed in this study are internationally recognized, non-proprietary clinical assessment methods routinely used in periodontal research and practice. These indices are available for research use without obtaining specific permission from the original developers, provided appropriate acknowledgment is made through citation of the relevant literature.

The examiner responsible for periodontal assessment was blinded to the salivary hs-CRP results, and laboratory personnel performing the ELISA assays were blinded to participants' clinical periodontal status.

Anthropometric assessment

Height and body weight were measured under standardized conditions using calibrated equipment, with participants barefoot and wearing light clothing. Body mass index (BMI) was calculated as weight (kg)/height² (m²) and treated as a continuous variable in the multivariable analyses.

Saliva collection and processing

To minimize circadian variability, unstimulated whole saliva specimens were obtained between 09:00 and 11:00 h. Participants abstained from food, beverages except water, toothbrushing, chewing gum, smoking, and mouthwash use for at least one hour before collection. After rinsing the mouth with distilled water, they remained seated at rest for approximately five minutes before saliva collection commenced.

Saliva was obtained using the passive drooling technique described by Navazesh [14]. Using the passive drool technique, saliva was allowed to accumulate naturally before being collected by expectoration into sterile polypropylene tubes. Collection continued for approximately 5-10 minutes, or until 3-5 mL of unstimulated saliva was obtained.

Immediately after collection, saliva samples were transported on ice to the laboratory and processed within 2 hours. Following centrifugation, the clarified supernatant was aliquoted into sterile microcentrifuge tubes and stored at −80°C until analysis. To minimize biomarker degradation, samples were not subjected to more than two freeze-thaw cycles. Before biochemical analysis, frozen aliquots were thawed at room temperature and centrifuged at 3000 × g for 5 minutes to remove precipitated proteins and residual particulate matter. Assay performance characteristics, including analytical sensitivity, lower limit of detection, and intra- and inter-assay coefficients of variation, were based on the manufacturer's validated specifications.

Estimation of salivary high-sensitivity CRP

Salivary hs-CRP concentrations were measured using a commercially available Human High-Sensitivity C-Reactive Protein (hs-CRP) ELISA Kit (Krishgen Biosystems Pvt. Ltd., Mumbai, India; Catalog No. KAPDB9001), according to the manufacturer's instructions. The assay had an analytical sensitivity of 0.01 ng/mL, a lower limit of detection of 0.02 ng/mL, an intra-assay coefficient of variation (CV) of < 8%, and an inter-assay CV of < 12%.A high-sensitivity ELISA was selected because salivary CRP concentrations are substantially lower than serum concentrations, necessitating an assay with enhanced analytical sensitivity for reliable quantification.

Before analysis, frozen saliva samples were thawed at room temperature and centrifuged at 3000 × g for five minutes to eliminate precipitated proteins. All saliva samples, standards, and quality-control specimens were analyzed in duplicate. Concentrations of hs-CRP were calculated from a standard calibration curve generated using a four-parameter logistic regression model. The analytical sensitivity, lower limit of detection, intra-assay coefficient of variation, and inter-assay coefficient of variation were documented according to the manufacturer's specifications. To minimize measurement bias, laboratory personnel performing the biochemical analyses remained blinded to the periodontal status of the study participants.

Statistical analysis

Statistical analyses were performed using IBM SPSS Statistics version 26.0 (IBM Corp., Armonk, NY, USA). Data distribution was evaluated using the Shapiro-Wilk test and confirmed by visual inspection of histograms and quantile-quantile (Q-Q) plots. Salivary hs-CRP concentrations were not normally distributed (Shapiro-Wilk p < 0.05) and were therefore log-transformed before inclusion in parametric analyses. Continuous variables are presented as mean ± standard deviation (SD) or median (interquartile range [IQR]) according to data distribution, whereas categorical variables are expressed as frequencies and percentages.

Salivary hs-CRP concentrations satisfied the assumptions for parametric analysis, including approximate normality and homogeneity of variance; therefore, group comparisons were performed using one-way ANOVA. Variables that violated distributional assumptions were analyzed using the Kruskal-Wallis test followed by Dunn's post hoc procedure.

Demographic, anthropometric, clinical, and biochemical characteristics were compared among the three study groups according to data distribution. Normally distributed variables were analyzed using one-way analysis of variance (ANOVA) with Bonferroni-adjusted pairwise comparisons, whereas non-normally distributed variables were compared using the Kruskal-Wallis test followed by Dunn's post hoc test. Categorical variables were analyzed using the chi-square test or Fisher's exact test, as appropriate.

The primary outcome was salivary high-sensitivity C-reactive protein (hs-CRP) concentration. Differences in hs-CRP concentrations among periodontally healthy participants and those with Stage I-II and Stage III-IV periodontitis were analyzed using one-way ANOVA or the Kruskal-Wallis test, with appropriate adjustment for multiple comparisons.

Associations between salivary hs-CRP concentrations and periodontal clinical parameters, including probing pocket depth (PPD), clinical attachment level (CAL), bleeding on probing (BOP), plaque index (PI), and gingival index (GI), were evaluated using Pearson's or Spearman's correlation coefficients according to data distribution.

Multiple linear regression was used to determine the independent association between salivary hs-CRP concentration and periodontitis severity. The regression model included age, sex, body mass index (BMI), and clinical stage of periodontitis as covariates. Model assumptions, including linearity, homoscedasticity, normality of residuals, independence of observations, and multicollinearity, were verified before analysis. Multicollinearity was assessed using the variance inflation factor (VIF), with values <5 considered acceptable.

Data completeness was assessed before statistical analysis. Participants with incomplete periodontal examination records or insufficient saliva samples were excluded from the final analysis. Because the proportion of missing data was minimal, no imputation procedures were applied.

Socioeconomic status, dietary habits, and oral hygiene behaviors were not included in the regression model because standardized quantitative measurements were not available for all participants; therefore, residual confounding from these variables cannot be completely excluded. All statistical tests were two-sided, and a p-value <0.05 was considered statistically significant.

## Results

Participant recruitment and study flow

**Figure 1:**
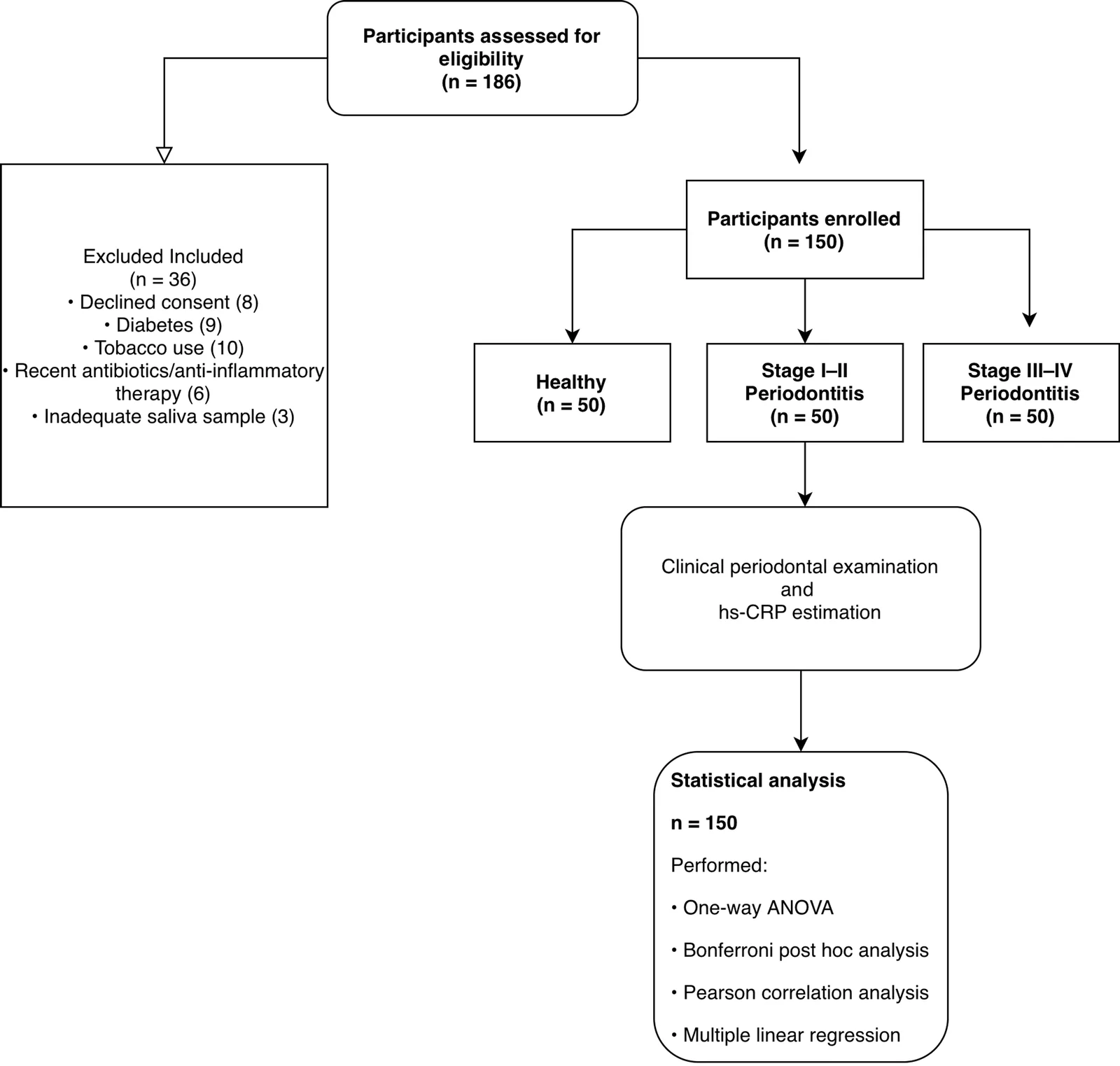
Study flow diagram The diagram summarizes participant screening, eligibility assessment, reasons for exclusion, allocation into the three study groups, and inclusion in the final statistical analysis. Abbreviations: STROBE: Strengthening the Reporting of Observational Studies in Epidemiology; hs-CRP: high-sensitivity C-reactive protein; ANOVA – Analysis of Variance

During the study period, 186 adults were screened for eligibility. Of these, 36 were excluded because they declined participation (n=8), had diabetes mellitus (n=9), were current tobacco users (n=10), had received antibiotics or anti-inflammatory medication within the predefined exclusion period (n=6), or provided insufficient saliva for biochemical analysis (n=3). The remaining 150 eligible participants were enrolled and allocated equally to three study groups: periodontally healthy (n=50), Stage I-II periodontitis (n=50), and Stage III-IV periodontitis (n=50). Clinical periodontal examination and salivary hs-CRP estimation were successfully completed for all enrolled participants, and complete datasets were available for the final analyses (Figure 1).

Baseline demographic, anthropometric, and periodontal characteristics of the study participants are summarized in Table 1. Participants with Stage III-IV periodontitis were significantly older than those in the periodontally healthy participants and Stage I-II groups (p < 0.001), whereas BMI did not differ significantly among the three groups (p=0.103). The proportion of male participants increased progressively with disease severity (p=0.047). All periodontal clinical parameters, including plaque index, gingival index, bleeding on probing, probing pocket depth, and clinical attachment level, demonstrated significant deterioration with increasing clinical stage of periodontitis (all p < 0.001). In addition, the number of natural teeth decreased significantly with disease severity (p < 0.001).

**Table 1:** Baseline demographic, anthropometric, and periodontal characteristics of the study participants according to clinical stage of periodontitis Values are presented as Mean ± SD for continuous variables and n (%) for categorical variables. Abbreviations: BMI: Body Mass Index; PI: Plaque Index; GI: Gingival Index; BOP: Bleeding on Probing; PPD: Probing Pocket Depth; CAL: Clinical Attachment Level; SD: Standard Deviation; ANOVA: Analysis of Variance; η²: Eta-squared; V: Cramér's V. Continuous variables were compared using one-way ANOVA, whereas categorical variables were compared using the Chi-square test. Degrees of freedom are presented in parentheses. Effect sizes: η² (0.01 = small, 0.06 = medium, 0.14 = large); Cramér's V (0.1 = small, 0.3 = medium, 0.5 = large). *Statistically significant (p < 0.05)

Variable	Periodontally healthy participants (n = 50)	Stage I–II (n = 50)	Stage III–IV (n = 50)	Test Statistic	Effect Size	p-value
Age (years), Mean ± SD	41.2 ± 11.3	47.3 ± 10.6	56.4 ± 9.8	F(2,147) = 18.42	η² = 0.20	<0.001*
Sex – Male, n (%)	22 (44.0)	30 (60.0)	34 (68.0)	χ²(2) = 6.12	V = 0.20	0.047*
Sex – Female, n (%)	28 (56.0)	20 (40.0)	16 (32.0)	—	—	—
BMI (kg/m²), Mean ± SD	23.7 ± 3.6	25.1 ± 4.0	25.0 ± 3.8	F(2,147) = 2.31	η² = 0.03	0.103
Number of natural teeth, Mean ± SD	27.4 ± 2.1	25.6 ± 3.4	21.8 ± 3.6	F(2,147) = 12.87	η² = 0.15	<0.001*
Plaque Index (PI), Mean ± SD	0.68 ± 0.24	1.54 ± 0.32	1.72 ± 0.38	F(2,147) = 25.84	η² = 0.26	<0.001*
Gingival Index (GI), Mean ± SD	0.52 ± 0.18	1.48 ± 0.35	1.56 ± 0.42	F(2,147) = 28.91	η² = 0.28	<0.001*
Bleeding on Probing (%), Mean ± SD	11.4 ± 7.2	42.6 ± 14.8	58.3 ± 16.5	F(2,147) = 32.47	η² = 0.31	<0.001*
Probing Pocket Depth (PPD, mm), Mean ± SD	1.8 ± 0.4	3.6 ± 0.8	5.2 ± 1.1	F(2,147) = 42.18	η² = 0.36	<0.001*
Clinical Attachment Level (CAL, mm), Mean ± SD	1.2 ± 0.5	3.1 ± 0.9	5.8 ± 1.3	F(2,147) = 48.63	η² = 0.40	<0.001*

**Figure 2:**
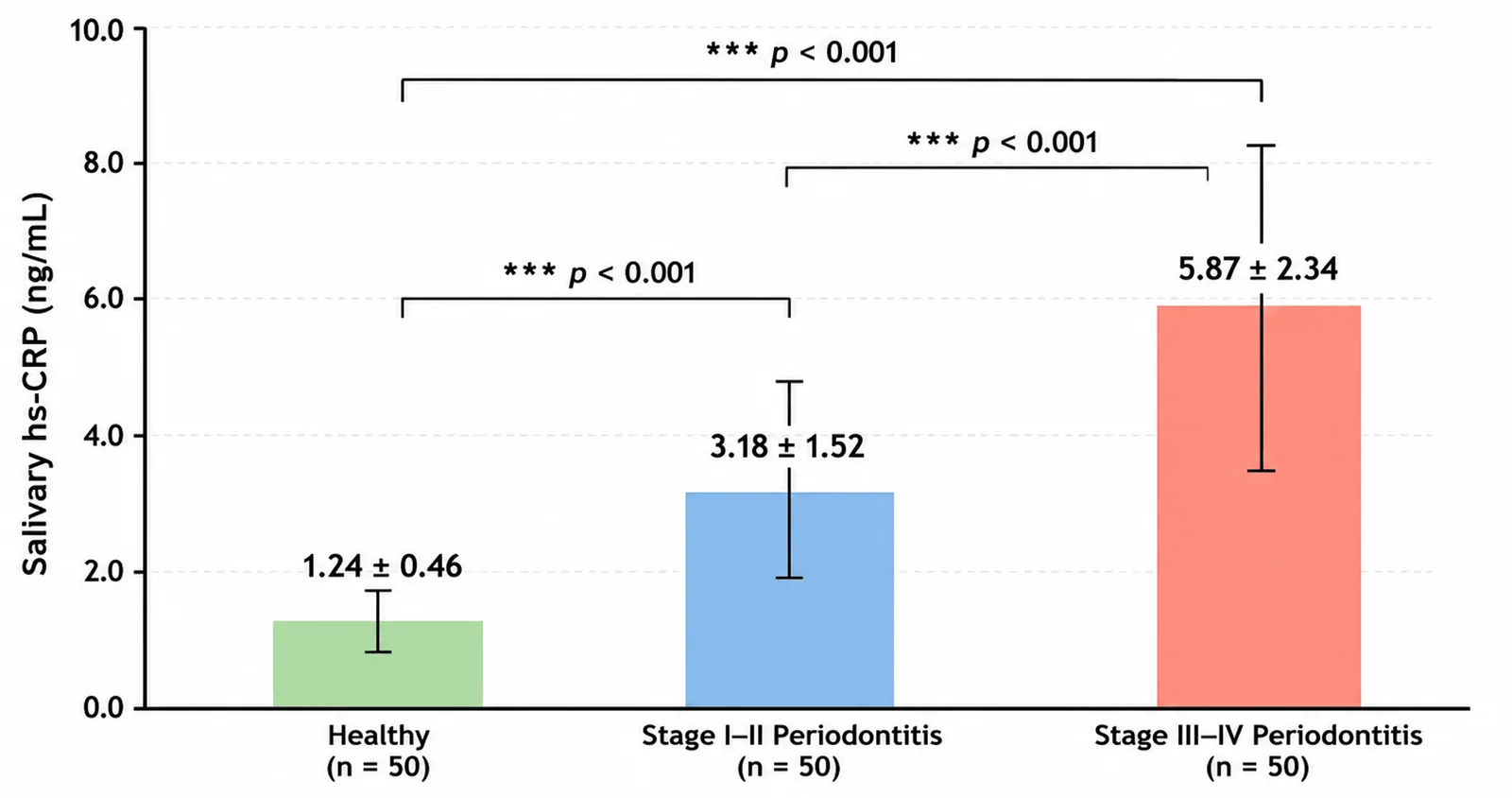
Mean salivary high-sensitivity C-reactive protein (hs-CRP) concentrations according to clinical stage of periodontitis. Bars represent the mean, and error bars indicate ± 1 standard deviation (SD). Group comparisons were performed using one-way analysis of variance (ANOVA) followed by Bonferroni post hoc analysis. Pairwise comparisons are indicated by brackets. ***p < 0.001 was considered statistically significant. Abbreviations: hs-CRP: high-sensitivity C-reactive protein; SD: standard deviation; ANOVA: analysis of variance.

Salivary hs-CRP concentrations increased progressively from periodontally healthy participants to Stage I-II and Stage III-IV periodontitis, as illustrated in Figure 2. The mean salivary hs-CRP concentrations according to clinical stage of periodontitis are presented in Table 2, with significantly higher levels observed in participants with more advanced disease (p < 0.001).

**Table 2:** Comparison of salivary high-sensitivity C-reactive protein (hs-CRP) concentrations among the study groups Values are presented as mean ± standard deviation (SD). Group comparisons were performed using one-way analysis of variance (ANOVA). Effect size is expressed as eta-squared (η²), where 0.01, 0.06, and ≥0.14 indicate small, medium, and large effects, respectively. Degrees of freedom are presented in parentheses. *Statistically significant (p < 0.05) Abbreviations: hs-CRP: high-sensitivity C-reactive protein; SD: Standard deviation; ANOVA: Analysis of variance; η²: eta-squared.

Variable	Periodontally healthy participants (n = 50)	Stage I–II (n = 50)	Stage III–IV (n = 50)	Test Statistic	Effect Size (η²)	p-value
Salivary hs-CRP (ng/mL), Mean ± SD	1.24 ± 0.46	3.18 ± 1.52	5.87 ± 2.34	F(2,147) = 36.74	0.33	<0.001*

Pairwise Bonferroni post hoc comparisons confirmed that all three study groups differed significantly in salivary hs-CRP concentrations (Table 3). 

**Table 3:** Bonferroni post hoc pairwise comparisons of salivary hs-CRP concentrations among the study groups Pairwise group comparisons were performed using the Bonferroni post hoc test following statistically significant one-way ANOVA. Mean differences are expressed as Healthy minus Stage I–II, Healthy minus Stage III–IV, and Stage I–II minus Stage III–IV, respectively. *Statistically significant (p < 0.05) Abbreviations: hs-CRP: high-sensitivity C-reactive protein; CI: Confidence interval; SE: standard error.

Comparison	Mean Difference	Standard Error	95% Confidence Interval	Adjusted p-value
Periodontally healthy participants vs Stage I–II	1.94	0.33	1.28–2.60	<0.001*
Periodontally healthy participants vs Stage III–IV	4.63	0.46	3.72–5.54	<0.001*
Stage I–II vs Stage III–IV	2.69	0.46	1.78–3.60	<0.001*

Correlation between salivary hs-CRP and periodontal parameters

Correlation analysis demonstrated significant positive associations between salivary hs-CRP concentrations and all periodontal clinical parameters, with the strongest correlations observed for clinical attachment level (CAL) and probing pocket depth (PPD) (Table 4). Moderate positive correlations were also identified for bleeding on probing (BOP) and gingival index (GI), whereas plaque index (PI) showed a comparatively weaker but statistically significant correlation. Among the demographic variables, age demonstrated a weak positive correlation with salivary hs-CRP concentrations, while body mass index (BMI) was not significantly correlated.

**Table 4:** Correlation between salivary hs-CRP concentrations and periodontal clinical parameters Correlation analysis was performed using Pearson's correlation coefficient. Abbreviations: hs-CRP: High-Sensitivity C-Reactive Protein; PI: Plaque Index; GI: Gingival Index; BOP: Bleeding on Probing; PPD: Probing Pocket Depth; CAL: Clinical Attachment Level; BMI: Body Mass Index; df: degrees of freedom. Effect size interpretation: r = 0.10 (small), 0.30 (medium), 0.50 (large). *Statistically significant (p < 0.05)

Variable	Correlation Coefficient (r)	Test Statistic (t)	Degrees of Freedom	p-value
Plaque Index (PI)	0.28	2.84	148	0.009*
Gingival Index (GI)	0.35	3.88	148	0.002*
Bleeding on Probing (%)	0.49	5.98	148	<0.001*
Probing Pocket Depth (PPD)	0.58	7.34	148	<0.001*
Clinical Attachment Level (CAL)	0.62	8.02	148	<0.001*
Age	0.27	2.98	148	0.008*
BMI	0.14	1.72	148	0.089

Multiple linear regression analysis

Multiple linear regression analysis was performed to identify independent factors associated with salivary hs-CRP concentrations after adjustment for age, sex, and body mass index (Table 5). Clinical stage of periodontitis remained independently associated with salivary hs-CRP concentrations, with both Stage I-II and Stage III-IV periodontitis demonstrating significantly higher hs-CRP concentrations than the healthy reference group (both p < 0.001). In contrast, age, sex, and BMI were not independently associated with salivary hs-CRP concentrations.

**Table 5:** Multiple linear regression analysis identifying independent predictors of salivary hs-CRP concentrations. Multiple linear regression analysis was performed with salivary hs-CRP concentration as the dependent variable. Independent variables included age, sex, body mass index, and clinical stage of periodontitis. Female sex and periodontally healthy participants served as the reference categories. Regression coefficients are presented as unstandardized beta coefficients (β) with corresponding standard errors (SE), standardized beta coefficients, 95% confidence intervals (CI), variance inflation factors (VIF), and p-values. VIF values <5 indicate no evidence of significant multicollinearity. *Statistically significant (p < 0.05) Abbreviations: hs-CRP: high-sensitivity C-reactive protein; β: regression coefficient; SE: standard error; CI, confidence interval; VIF, variance inflation factor.

Predictor Variable	Unstandardized β	Standard Error	Standardized β	t Statistic	95% Confidence Interval	VIF	p-value
Age (years)	0.06	0.03	0.14	1.79	−0.01 to 0.13	1.24	0.076
Sex (Male)	0.32	0.33	0.06	1.02	−0.33 to 0.97	1.08	0.312
Body Mass Index (kg/m²)	0.05	0.04	0.08	1.28	−0.03 to 0.13	1.12	0.204
Stage I–II Periodontitis	1.68	0.38	0.28	4.42	0.92 to 2.44	1.41	<0.001*
Stage III–IV Periodontitis	3.82	0.46	0.51	8.31	2.91 to 4.73	1.38	<0.001*

The overall regression model was statistically significant (Table 6) and explained approximately 48% of the variability in salivary hs-CRP concentrations (R² = 0.482; adjusted R² = 0.462; p < 0.001). All variance inflation factor (VIF) values were below 2.0, indicating no evidence of clinically relevant multicollinearity among the predictor variables.

**Table 6:** Summary of the multiple linear regression model Model performance was assessed using multiple linear regression. The coefficient of determination (R²) represents the proportion of variance in salivary hs-CRP concentrations explained by the independent variables included in the model. Statistical significance of the overall regression model was evaluated using the F-test. *Statistically significant (p < 0.05) Abbreviations: R²: coefficient of determination.

Parameter	Value
Coefficient of determination (R²)	0.482
Adjusted R²	0.462
F Statistic	F(5,144) = 18.24
Model p-value	<0.001*

## Discussion

Progressively higher salivary hs-CRP concentrations were observed with increasing periodontitis severity. Multivariable regression analysis confirmed that this association was independent of age, sex, and body mass index. These findings may represent a promising adjunctive biomarker; however, its diagnostic performance has not yet been established because this study did not evaluate sensitivity, specificity, receiver operating characteristic (ROC) characteristics, likelihood ratios, or predictive values.The graded increase in salivary hs-CRP observed across periodontal stages suggests that worsening periodontal destruction is accompanied by an enhanced inflammatory response, supporting saliva as a reflection of both local and systemic inflammation [2-5,15].

The observed increase in salivary hs-CRP concentrations is biologically consistent with the chronic inflammatory process underlying periodontitis. Continuous exposure to periodontal biofilm activates host immune pathways, promoting the release of inflammatory mediators, including IL-1β, IL-6, and TNF-α. Systemic release of these cytokines, particularly interleukin-6, stimulates hepatic synthesis of CRP, while increased vascular permeability and gingival crevicular fluid flow facilitate the transfer of inflammatory proteins into the oral cavity, resulting in elevated salivary hs-CRP concentrations [3,5,8]. Recent evidence suggests that salivary inflammatory biomarkers reflect both local periodontal inflammation and systemic inflammatory burden, supporting their potential role as non-invasive indicators of periodontal disease activity and progression [5,16,17].

The present findings are consistent with previous investigations demonstrating significantly higher CRP concentrations among patients with periodontitis compared with periodontally healthy individuals [2,4,7,9,10]. Published systematic reviews and meta-analyses consistently demonstrate higher circulating CRP concentrations in patients with periodontitis, with reductions observed after periodontal treatment, suggesting that periodontal inflammation influences systemic inflammatory activity [3,4]. Although many earlier studies focused primarily on serum CRP, the present study extends these observations by demonstrating comparable trends using saliva. Because saliva collection is non-invasive, inexpensive, and easily repeatable without venipuncture, it offers several practical advantages over blood-based testing for disease screening, monitoring therapeutic response, and large-scale community-based periodontal surveillance [5,16,18].

Unlike several earlier studies that employed the traditional chronic/aggressive periodontitis classification, the present study adopted the contemporary 2017 World Workshop Classification, which emphasizes disease staging and grading. Consequently, the findings are directly applicable to current periodontal diagnostic practice and extend existing evidence within a modern classification framework. This approach facilitates greater clinical relevance and comparability with contemporary periodontal research while reflecting current international diagnostic recommendations [8].

An important finding of the present study was the significant positive association between salivary hs-CRP concentrations and established periodontal clinical parameters. The strongest correlations were observed with clinical attachment level and probing pocket depth, whereas moderate correlations were identified with bleeding on probing and gingival index. In contrast, plaque index demonstrated a comparatively weaker association with hs-CRP concentrations. These findings suggest that salivary hs-CRP reflects cumulative periodontal tissue destruction and inflammatory burden rather than simply the presence of dental plaque. Similar relationships have been reported previously, in which CRP concentrations increased proportionally with worsening periodontal destruction and clinical disease severity [4,5,9,10]. The stronger association with clinical attachment loss further supports the potential utility of salivary hs-CRP as a biomarker reflecting disease severity rather than isolated plaque-induced inflammation.

Multiple linear regression analysis further demonstrated that clinical stage of periodontitis remained an independent predictor of salivary hs-CRP concentrations after adjustment for age, sex, and body mass index. In contrast, none of the demographic variables independently influenced hs-CRP concentrations. These findings suggest that the elevated inflammatory burden observed in the study population was primarily attributable to periodontal disease severity rather than demographic characteristics. Similar observations have been reported in recent longitudinal biomarker studies, where periodontal inflammation remained independently associated with inflammatory biomarker profiles after controlling for potential confounding factors [5,6]. Furthermore, the coefficient of determination (R² = 0.482) indicates that nearly half of the variability in salivary hs-CRP concentrations was explained by the variables included in the regression model, highlighting periodontal disease severity as an important determinant of salivary inflammatory burden.

From a clinical perspective, these findings support the growing interest in saliva-based diagnostics for periodontal disease. Recent advances in salivary proteomics, multiplex biomarker panels, and point-of-care diagnostic technologies have further strengthened the potential clinical utility of saliva for periodontal diagnosis, disease monitoring, and precision periodontal care [16-18]. Nevertheless, because hs-CRP is a non-specific inflammatory biomarker, elevated concentrations may also occur in systemic inflammatory conditions such as cardiovascular disease, diabetes mellitus, autoimmune disorders, and acute infections [3,4]. Therefore, salivary hs-CRP should be interpreted as an adjunct to comprehensive periodontal examination and complementary salivary biomarkers rather than as a standalone diagnostic test [17,18].

The results should be interpreted within the context of the study design. Owing to its cross-sectional nature, the investigation identifies associations between periodontal inflammation and salivary hs-CRP concentrations but cannot establish temporal or causal relationships. Third, only one inflammatory biomarker was investigated, whereas incorporation of additional cytokines, matrix metalloproteinases (MMPs), or proteomic biomarkers may improve diagnostic accuracy. Nevertheless, the study included clinically well-characterized participants representing the full spectrum of periodontal disease severity and employed multivariable regression analysis to minimize the influence of important confounding variables. Although important systemic inflammatory conditions were excluded during participant selection, residual confounding from unmeasured factors that may influence salivary hs-CRP concentrations cannot be completely excluded. As recruitment was limited to one tertiary care center, caution should be exercised when extrapolating these findings to other populations or clinical settings.

Participants were recruited consecutively from a single tertiary care hospital, which may have introduced selection bias and limits the representativeness of the study population. Furthermore, exclusion of smokers, obese individuals, and patients with systemic inflammatory diseases enhanced internal validity by minimizing confounding but may reduce the generalizability of the findings to routine periodontal practice. Salivary hs-CRP concentrations were measured at a single time point; therefore, intra-individual biological variability and temporal fluctuations could not be assessed. Finally, because periodontal staging incorporates clinical attachment level and probing pocket depth, the observed correlations between hs-CRP concentrations and these parameters should be interpreted cautiously, as partial structural correlation is inherent within the classification framework.

Future investigations should evaluate salivary hs-CRP alongside complementary biomarkers to determine whether multimarker approaches enhance diagnostic accuracy. Validation across independent multicenter cohorts will be essential before such biomarker panels can be translated into routine clinical assessment of periodontitis [16-18].

## Conclusions

This study demonstrated a significant and progressive increase in salivary high-sensitivity C-reactive protein (hs-CRP) concentrations with advancing clinical stage of periodontitis in an Indian population. The association between salivary hs-CRP concentration and periodontal severity remained evident after controlling for age, sex, and body mass index. These findings support the potential role of salivary hs-CRP as a promising adjunctive biomarker; however, its diagnostic accuracy and incremental clinical utility require confirmation through dedicated diagnostic accuracy and prospective longitudinal studies before routine clinical implementation can be recommended. Although salivary hs-CRP should not be considered a standalone diagnostic marker because of its non-specific inflammatory nature, its ease of collection and patient acceptability make it a promising candidate for incorporation into saliva-based diagnostic strategies. Further large-scale, multicenter prospective studies are warranted to validate these findings, evaluate longitudinal changes following periodontal therapy, and determine the incremental diagnostic value of hs-CRP when combined with other salivary biomarkers in multimarker panels for precision periodontal care.
